# Pulmonary aspiration during pregnancy or immediately postpartum in the UK: A population-based case-control study

**DOI:** 10.3310/nihropenres.13797.1

**Published:** 2025-01-08

**Authors:** Nuala Lucas, Alison Gooda, Ruth Tunn, Marian Knight, The UKOSS Aspiration in Pregnancy investigators, Vikash Mistry, David Bogod, Audrey Quinn, Kim Hinshaw

**Affiliations:** 1 London North West Healthcare NHS Trust, Watford Road, Harrow, Middlesex, HA1 3UJ, UK; 2 National Perinatal Epidemiology Unit, University of Oxford, Old Road Campus, Headington, Oxford, OX3 7LF, UK

**Keywords:** pulmonary aspiration, incidence, pregnancy, surveillance, UK Obstetric Surveillance System

## Abstract

**Background:**

Pulmonary aspiration of gastric contents is the most frequent cause of death associated with complications of airway management during general anaesthesia. Pregnancy increases aspiration risk owing to factors including delayed gastric emptying and increased intragastric pressure. We describe the incidence, risk factors, management, and outcomes of maternal pulmonary aspiration in pregnancy in the UK.

**Methods:**

We conducted a population-based surveillance and case-control study. Between September 2013 and August 2016, all UK consultant-led obstetric units prospectively identified cases of pulmonary aspiration among parturient women using a pre-defined case definition, and reported them via the UK Obstetric Surveillance System (UKOSS). Controls (n=1982) were obtained from four UKOSS studies conducted between 2005 and 2014. We calculated the incidence of pulmonary aspiration using 2013–2015 maternities as the denominator. We explored potential risk factors for aspiration using univariable logistic regression and described outcomes.

**Results:**

We identified 12 cases of pulmonary aspiration, giving an incidence of 5.2 per 1,000,000 maternities (95% CI 2.69-9.09). Cases were significantly less likely than controls to be multiparous (unadjusted odds ratio [uOR] 0.255, 95% CI 0.069-0.946), and significantly more likely to undergo caesarean section (uOR 24.89, 95% CI 3.18-194.85) and to receive general anaesthetic for caesarean section (p<0.001). Gestation was significantly shorter in cases than controls (uOR 0.782, 95% CI 0.702-0.870). Women who aspirated were significantly more likely to be admitted to the intensive therapy unit than controls (p<0.001). Infants of women who aspirated had significantly lower Apgar scores and were more likely to be admitted to the neonatal intensive care unit or to be stillborn compared with infants of women in the control group.

**Conclusions:**

Pulmonary aspiration is rare in UK obstetric anaesthetic practice; however, it remains a risk of general anaesthesia. Despite a large study population, our analyses lacked power to evaluate many potential risk factors. Future research should focus on developing methods to accurately identify pregnant women at risk of aspiration.

## Plain Language Summary

Pulmonary aspiration is a condition in which stomach contents are inhaled into the windpipe and lungs. This most commonly occurs in patients under general anaesthesia, and can be fatal. Pregnancy increases the risk of pulmonary aspiration because of physical and hormonal changes it causes in the digestive system. Therefore, pregnant women giving birth under general anaesthetic are at particularly high risk.

We wanted to understand how common pulmonary aspiration is among pregnant women in the UK, and how women affected by this condition differ from other women. We also investigated how the condition was managed, and how affected women and their babies fared.

The UK Obstetric Surveillance System (UKOSS) collects information from all UK maternity units about pregnant women with certain conditions. We used UKOSS to collect anonymous information about all women who experienced pulmonary aspiration during a three-year period. We compared these women with pregnant women who did not experience pulmonary aspiration.

During the three years, 12 pregnant women in the UK experienced pulmonary aspiration when giving birth. Based on this, we estimated that approximately 5.5 women were affected per million women giving birth. As expected, women who aspirated were more likely than unaffected women to give birth by caesarean section under general anaesthesia. They were also more likely to be first-time mums, and to give birth earlier (at 37.6 weeks on average, compared with 39 weeks for unaffected women).

The results of our study suggest that pulmonary aspiration is reassuringly uncommon among women giving birth in the UK, including among the higher-risk group of women giving birth under general anaesthesia. However, when it did occur, women were more likely to need to be admitted to intensive care; and the babies of affected women typically needed additional medical support and there was an increased risk of stillbirth. Better methods are needed to identify women who are at risk of pulmonary aspiration.

## Introduction

Pulmonary aspiration of gastric contents is a serious potential complication of anaesthesia and was the most frequent cause of death in cases reported to the Royal College of Anaesthetists 4th National Audit Project (NAP4) ^1^. Pregnant women are considered at increased risk of aspiration as a result of the hormonal and physical changes of pregnancy and labour. Increased progesterone levels lead to a reduction in the lower oesophageal sphincter tone and delayed gastric emptying, and there is an increase in intragastric pressure as a result of the gravid uterus ^2^. Besides pregnancy, identified risk factors for pulmonary aspiration of gastric contents include full stomach, hiatus hernia, dyspepsia, morbid obesity, receipt of opioids, and use of first-generation supraglottic airway devices ^3, 4^.

Since Mendelson combined observational evidence with animal experiments to describe his eponymous syndrome of acid aspiration pneumonitis in 1946 ^5^, measures to reduce morbidity and mortality from aspiration have been introduced to obstetric practice. These include restriction of oral intake in labour, use of H2-receptor drugs and sodium citrate to reduce gastric volume and acidity, airway protection with rapid sequence induction involving pre-oxygenation, cricoid pressure, use of succinylcholine, and avoidance of mask ventilation before tracheal intubation ^2, 6^. Despite these measures, aspiration remains a potential complication of obstetric anaesthesia.

Although restriction of food intake during labour as a measure to prevent aspiration is common and is recommended in American ^7^ and European ^8, 9^ national guidelines, available evidence does not support this practice ^10^. Alongside these concerns is the desire to maximise a woman’s comfort in labour. In 2007, NICE guidance on intrapartum care was changed to advise a light diet in established labour, as long as women have not received opioids and are not at high risk of requiring an emergency caesarean birth ^11^.

The comparative rarity of obstetric pulmonary aspiration makes it challenging to estimate its incidence. Studies that have attempted to do so to date have mainly focused on the higher-risk population of women undergoing general anaesthesia. A prospective observational study in Australia confirmed one case of aspiration in 1095 women who underwent caesarean section under general anaesthesia, suggesting an incidence of 0.1% (95% CI 0.002%–0.5%) in this population ^12^, while a German single-centre retrospective study of 2,390 obstetric surgery patients at considered at high risk of pulmonary aspiration did not detect any aspiration events ^13^. A UKOSS study of failed intubation during obstetric anaesthesia estimated a higher incidence of pulmonary aspiration of 8% in cases of failed intubation and 1% in matched controls (pregnant women from the same hospitals who underwent general anaesthesia and successful intubation) ^14^.

The aims of this study were to use the UK Obstetric Surveillance System (UKOSS) ^15^ to calculate the national incidence of pulmonary aspiration in obstetrics, to identify the risk factors for pulmonary aspiration in obstetrics in the UK, and to describe how pulmonary aspiration is currently managed in the UK.

## Methods

### Patient and Public Involvement

Patients were not directly involved in the design of the study. Two members of the public were indirectly involved in the design of the study via representatives on the UKOSS Steering Committee, which reviews, comments on, and approves all studies to be run through UKOSS.

A national, population-based prospective surveillance and case-control study was undertaken using UKOSS. UKOSS is designed to study rare and severe complications of pregnancy and childbirth in the UK and is now well established, having started data collection in 2005 ^15^. Nominated clinicians at each obstetrician-led maternity unit in the UK completed a monthly return to UKOSS. This return notified UKOSS of any cases of pulmonary aspiration that occurred between September 1, 2013 and August 31, 2016. A data collection form was then sent to any unit reporting a case to collect more detailed information. The data collection form sought confirmation of the appropriate case definition and additional information on risk factors, management, and outcomes. The full form is available at https://www.npeu.ox.ac.uk/assets/downloads/ukoss/forms/UKOSS-Aspiration-in-Pregnancy.pdf. It was developed specifically for reporting cases of pulmonary aspiration and designed to be short and easily completed from a woman's case notes without requiring reference to any other sources of information. The co-investigator group developed the form and crosschecked with anaesthetists in a series of revisions to ensure that the questions thoroughly covered the details required for the study.

The case definition was based on the findings of NAP4 ^1^ and included women at 20 weeks’ gestation or greater who had an unprotected airway while unconscious, semiconscious or paralysed as well as a clinical history consistent with regurgitation of stomach contents and pulmonary aspiration, such as vomiting or gastric contents seen in the oropharynx. This must have been accompanied by signs of respiratory compromise requiring supplementary oxygen and antibiotics or level 2/3 respiratory support without any other apparent cause. To minimise missed cases, the Mothers and Babies: Reducing Risk through Audits and Confidential Enquiries across the UK (MBRRACE-UK) maternal mortality reports for the surveillance period of this study were crosschecked for additional aspiration cases.

Unmatched controls were obtained from four UKOSS studies conducted between 2005 and 2014 ^16– 19^, defined as the two women giving birth before the notified case in these studies. This gave a total of 1982 women without severe life-threatening complications throughout pregnancy or childbirth.

### Statistical analysis

Incidence of pulmonary aspiration with 95% confidence interval (CI) was calculated using the national surveillance data. Denominators were total maternities in the UK between 1 January, 2013 and 31 December, 2015, obtained from the Office for National Statistics (ONS) for England and Wales ^20^, National Records of Scotland (NRS) ^21^, and Northern Ireland Statistics and Research Agency (NISRA) ^22^. The characteristics of cases and controls were compared using Chi-Square test and Fisher's Exact test for difference in proportions for categorical variables. Medians and interquartile ranges were calculated for continuous variables (maternal age, BMI, and gestational weeks at delivery) for cases and controls, then compared using Wilcox rank-sum test, as they were found to be non-normally distributed. Because it is thought to increase the risk of difficult tracheal intubation ^23^, obesity was examined as a categorical variable (obese, BMI ≥30 vs non-obese, BMI <30) in addition to BMI being evaluated as a continuous variable.

Univariable logistic regression analysis was conducted to examine the association of potential risk factors individually with pulmonary aspiration. Owing to the small number of events, multivariable logistic regression analysis was not undertaken to prevent bias due to multiple testing. Missing values were excluded from these calculations owing to their small number.

Case characteristics for which control data were not available (medical history specifically relevant to pulmonary aspiration, observed clinical signs of aspiration, setting of aspiration, X-ray consolidation, X-ray abnormalities, presence of consultant anaesthetist, oral intake, antacid prophylaxis, use of opioid, use of airway device and mechanisms to reduce risk) are described.

Maternal and infant outcomes (maternal ITU admission, stillbirth, infant death, infant neonatal intensive care admission and infant Apgar score) were compared between cases and controls using Chi-Square/Fisher's Exact tests.

Associations were considered significant at a p-value of <0.05 (two-sided). All analyses were undertaken using STATA version 13.1, SE ^24^.

### Study power

Based on the final data, the study had 80 percent power (α=0.05) to detect odds ratios (ORs) between 0.39 and 4.55 for the most prevalent (multiparity) and least prevalent (multiple pregnancy) risk factors amongst the control group, respectively.

## Results

Over the three years of surveillance, there were 12 confirmed cases of pulmonary aspiration out of a total of 2,305,920 maternities ^20– 22^. Data (including zero-value notifications) were received from all UK consultant-led maternity units and no missed cases were identified via crosschecking with the MBRRACE-UK reports. This gives an estimated incidence of 5.2 cases per 1,000,000 maternities (95% CI 2.69–9.09), equating to 1 in 192,309 maternities or 0.00052%. There were no deaths resulting from pulmonary aspiration; one woman in the case group died from other causes. There were four cases in year one, five in year two and three in year three, with no statistically significant trend within this time period (p=0.72).

Of the 12 cases, aspiration in nine was associated with general anaesthesia: seven for caesarean section and two for surgical management of miscarriage and cardiac arrest respectively. The three non-general anaesthesia-associated cases of aspiration occurred in association with 1) recurrent epileptic fits, 2) cardiac problems during pregnancy and 3) in a semiconscious patient with obtunded airway reflexes.

The characteristics of cases and controls are shown in Table 1. The odds of aspiration were higher in obese women (uOR 1.59, 95% CI 0.410–6.182), but this difference was not statistically significant (p=0.502). The odds of aspiration were also higher in women who had caesarean sections (uOR 24.89, 95% CI 3.18–194.85, p=0.002). Of those patients who underwent a caesarean section, cases were statistically significantly more likely have undergone general rather than regional anaesthesia (p<0.001). Multiparous women were less likely to aspirate (uOR 0.255, 95% CI 0.069–0.946). Median duration of gestation was significantly shorter in cases (37.6 weeks, interquartile range (IQR) 33–40.4 weeks) than in controls (39 weeks, IQR 38–40 weeks; uOR 0.782, 95% CI 0.702–0.870).

**Table 1.** Comparison of characteristics of cases and controls.

*Category*	Risk Factor	Cases (N = 12 unless otherwise stated)	Controls (N = 1982 unless otherwise stated)	p-value *	uOR	uOR p-value	95% CI
*Sociodemographic * *factors*	**Ethnicity, n (%)**			1.000			
	White	10 (83)	1540 (78)		1		
	Non-white	2 (16)	424 (21)		0.726	0.681	0.159–3.328
	Unknown	0 (0)	18 (0.9)				
	**Age, median (IQR)**	30.5 (27–33.5)	29 (25–34)	0.565	1.03	0.518	0.940–1.131
	**Number of fetuses, n (%)**			0.177			
	1	11 (92)	1951 (98)		1		
	2	1 (8)	31 (2)		5.721	0.100	0.716–45.689
	**Employment, n (%)**			0.304			
	Employed	9 (75)	1168 (59)		1		
	Unemployed	1 (8)	434 (22)		0.299	0.253	0.038–2.367
	Unknown	2 (17)	380 (19)				
*Medical and * *obstetric factors*	**BMI (kg/m ^2^), median (IQR)**	27.95 (24.5–35.4)	24.6 (21.9–29)	0.070	1.08	0.091	0.988–1.181
	**Obesity, n (%)**			0.451			
	Obese (BMI ≥30)	3 (25)	409 (21)		1.59	0.502	0.410–6.182
	Non-obese (BMI <30)	7 (58)	1519 (77)		1		
	Unknown	2 (17)	54 (3)				
	**Gestation (weeks), median (IQR)**	37.6 (33–40.4)	39 (38–40)	0.044	0.782	<0.001	0.702–0.870
	**Parity, n (%)**			0.038			
	Multiparous	3 (25)	1119 (56)		0.255	0.041	0.069–0.946
	Nulliparous	9 (75)	857 (43)		1		
	Unknown	0 (0)	6 (0.3)				
*Parturition*	**Labour induction, n (%)**			0.497			
	Yes	4 (33)	525 (26)		1.58	0.467	0.461–5.421
	No	7 (58)	1452 (73)		1		
	N/A	1 (8)	5 (0.3)				
	**Mode of delivery, n (%)**						
	Spontaneous vaginal	0 (0)	1138 (57)				
	Operative vaginal	1 (8)	266 (13)				
	Breech	0 (0)	3 (0.2)				
	Pre-labour C-section	4 (33)	339 (17)				
	C-section after labour onset	6 (50)	228 (12)				
	N/A (case) / missing (control)	1 (8)	8 (0.4)				
	**C-section, n (%)**			<0.001			
	Yes	10 (83)	567 (29)		24.89	0.002	3.18–194.85
	No	1 (8)	1411 (71)		1		
	N/A (case) / missing (control)	1 (8)	4 (0.2)				
	**Anaesthesia method for** ** C-section, n (%)**	**N=10**	**N=567**	<0.001			
	Regional	0 (0)	527 (93)				
	General	9 (90)	36 (6)				
	Unknown	1 (10)	4 (1)				

*Calculated without missing values, using Fisher’s exact test for categorical variables and Wilcox rank-sum test for continuous variables IQR, interquartile range; BMI, body mass index; uOR, unadjusted odds ratio; CI, confidence interval; C-section, caesarean section. N/A, not applicable (pregnancy ended in miscarriage)

Additional characteristics of cases are presented in Table 2. Two of the twelve women had epilepsy, one had swallowing problems resulting from tracheoesophageal surgery, and one had a respiratory illness. Prior to the aspiration event, low oxygen saturation was observed in seven of the women. Gastric contents were detected in the oropharynx of six of the women, and four women vomited prior to aspiration.

**Table 2.** Non-comparable case characteristics.

Characteristic	n (%) (N =12 unless otherwise stated)
**Relevant medical history**	
Hiatus hernia	0 (0)
History of aspiration	0 (0)
Swallowing problems	1 (8)
GORD	0 (0)
Epilepsy	2 (17)
Respiratory illness	1 (8)
**Observed features before aspiration**	
Vomiting	4 (33)
Gastric contents oropharynx	6 (50)
Gastric contents pillow	1 (8)
Low O _2_ sat	7 (58)
UA induction/ intubation GA	2 (17)
UA extubation after GA	2 (17)
UA while semiconscious	3 (25)
**Place of aspiration**	
Labour ward theatre	7 (58)
Labour ward recovery area	1 (8)
Other within hospital	4 (33)
**X-ray consolidation**	
Yes	7 (58)
No	5 (42)
**Other x-ray abnormality**	
Yes	4 (33)
No	8 (67)
**Presence of consultant anaesthetist**	**N=9**
Consultant administered GA	4 (44)
Consultant present at GA	1 (11)
No consultant	4 (44)

GORD, gastro-oesophageal reflux disease; UA, upper airway; GA, general anaesthesia

Pre-aspiration management of the cases is shown in Table 3. Seven of the 12 cases (58%) were known to have had oral intake during the 6 hours preceding the aspiration event: three food and fluid, one fluid, and three clear fluid only. Seven of the 12 cases (58%) had received antacid prophylaxis in the 6 hours preceding the aspiration event: six women had received ranitidine, three sodium citrate, and two metoclopramide. Six of the 12 women (50%) had received opioids. Airway devices were in place in six patients at the time of aspiration; these included one oropharyngeal airway, two supraglottic airway devices and two cuffed tracheal tubes. One woman aspirated following oesophageal intubation. Of the nine cases in whom aspiration was thought to be associated with general anaesthesia, seven (78%) received protective cricoid pressure. In women in whom aspiration occurred at the end of surgery and anaesthesia, none was extubated in the left lateral position.

**Table 3.** Pre-aspiration management of cases.

Procedure	n (%) (N = 12 unless otherwise stated)
**Oral intake in last 6 hrs**	
Yes	7 (58)
*Food and fluids*	*3 (43)*
*Fluids only*	*1 (14)*
*Clear fluids only*	*3 (43)*
No	2 (17)
Unknown	3 (25)
**Antacid prophylaxis in last 6 hours**	
Yes	7 (58)
*Ranitidine*	*6 (86)*
*Sodium citrate*	*3 (43)*
*Metoclopramide*	*2 (29)*
No	5 (42)
**Opioid in last 6 hrs**	
Yes	6 (50)
*Morphine*	*1 (17)*
*Fentanyl*	*3 (50)*
*Alfentanil*	*1 (17)*
*Codeine*	*1 (17)*
No	6 (50)
**Airway device in place**	
Yes	6 (50)
*Oropharyngeal airway*	*1 (17)*
*Classic laryngeal mask airway*	*0 (0)*
*Other supraglottic*	*2 (33)*
*Endotracheal tube cuff inflated*	*2 (33)*
*Endotracheal tube cuff not inflated*	*0 (0)*
*Other*	*1 (17)*
No	1 (8)
Unknown	3 (25)
**Mechanism to reduce aspiration ** **risk if aspiration thought to be ** **associated with GA**	**N=9**
Cricoid pressure	7 (78)
Extubated in left lateral position	0 (0)

GA, general anaesthesia

Details of case management following the aspiration event are presented in Table 4. Six cases (50%) underwent tracheal intubation, four (33%) received cricoid pressure, and seven (58%) had their oropharynx suctioned to prevent further aspiration. Four patients (33%) were placed in the left lateral, head down position, and ten (83%) received supplementary oxygen. Bronchoscopy was performed on one woman. All of the cases received antibiotics, with co-amoxiclav (n=5), metronidazole (n=5), and tazocin (n=4) being the most common. Only two women (17%) received steroids (budesonide/formoterol and hydrocortisone).

**Table 4.** Post-aspiration management of cases.

Intervention	n (%) (N = 12)
**Intubation**	6 (50)
**Cricoid pressure**	4 (33)
**Oropharyngeal suction**	7 (58)
**Left-lateral position**	4 (33)
**Supplementary oxygen**	10 (83)
**Bronchoscopy**	1 (8)
**Antibiotic given**	
Yes	12 (100)
*Co-amoxiclav*	*5 (42)*
*Amoxicillin*	*1 (8)*
*Tazocin*	*4 (33)*
*Cefuroxime*	*3 (25)*
*Meropenem*	*1 (8)*
*Clindamycin*	*1 (8)*
*Metronidazole*	*5 (42)*
*Gentamycin*	*3 (25)*
No	0 (0)
**Steroids given**	
Yes	2 (17)
*Symbicort*	*1 (50)*
*Hydrocortisone*	*1 (50)*
No	10 (83)
**Bronchodilator given**	
Yes	1 (8)
*Salbutamol*	*1 (100)*
No	11 (92)

Maternal and infant outcome measures for cases and controls are presented in Table 5. Women who aspirated were statistically more likely to be admitted to the ITU (p<0.001). Infants of women who aspirated had significantly lower Apgar scores (p=0.003) and were more likely to be admitted to the NICU (p<0.001) or to be stillborn (p=0.048) compared with infants born to women in the control group.

**Table 5.** Maternal and neonatal clinical outcomes.

*Category*	Outcome	Cases	Controls	p-value *
*Maternal*		**N = 12**	**N= 1982**	
	**Maternal ITU admission, n (%)**			<0.001
	Yes	9 (75)	23 (1)	
	No	3 (25)	1955 (99)	
	Unknown	0 (0)	4 (0.2)	
	Length of ITU stay (days), median (IQR)	2 (1–4)		
	**Maternal death, n (%)**	1 (8)	0 (0)	
	**HDU admission, n (%)**			
	Yes	5 (42)		
	No			
	Length of HDU stay (days), median (IQR)	2 (1–3)		
	**Miscarriage, n (%)**			
	Yes	1 (8)		
	No	11 (92)		
	Unknown	0 (0)		
	**Morbidity, n (%)**			
	Mechanical ventilation	6 (50)		
	ECMO	1 (8)		
	Cardiorespiratory arrest	2 (17)		
	Resuscitation required	6 (50)		
	**Length hospital stay post aspiration ** **(days), median (IQR)**	8 (5–18)		
*Infant*	**Stillbirth, n (%)**	N = 12 **	**N = 2013**	0.048
	Yes	1 (8)	8 (0.4)	
	No	11 (92)	1996 (99)	
	Unknown	0 (0)	9 (0.4)	
	**Infant death, n (% of live births)**	**N = 11**	**N = 2005**	1.000
	Yes	0 (0)	4 (0.2)	
	No	11 (100)	1989 (99)	
	Unknown	0 (0)	12 (0.6)	
	**NICU admission, n (%)**	**N = 11**	**N = 2005**	<0.001
	Yes	6 (55)	147 (7)	
	No	4 (36)	1846 (92)	
	Unknown	1 (9)	12 (0.6)	
	**Apgar score, median (IQR)**	8 (5–9)	9 (9–10)	0.003

*Calculated without missing values, using Fisher’s exact test for categorical variables and Wilcox rank-sum test for continuous variables **12 babies born to 11 mothers ITU, intensive therapy unit; HDU, high-dependency unit; IQR, interquartile range; ECMO, extracorporeal membrane oxygenation; NICU, neonatal intensive care unit

## Discussion

The incidence of pulmonary aspiration in obstetric patients in the UK is very low, with an incidence estimated by this study of 5.2 per 1,000,000 maternities (one in 192,309 maternities or 0.00052%). No significant temporal trend was found in the study's timeframe. Duration of gestation, parity, caesarean delivery, and general anaesthesia for caesarean section were all statistically significantly associated with the occurrence of pulmonary aspiration.

Pulmonary aspiration of gastric contents was a significant cause of anaesthesia-related mortality during the early years of the UK Maternal Confidential Death Enquiries, which began in the early 1950s. In the 1950s and 1960s, aspiration accounted for a substantial proportion of maternal deaths, highlighting the need for better airway management and preoperative fasting guidelines ^25, 26^. Improved training, advancements in anaesthetic techniques such as endotracheal intubation, and the introduction of cricoid pressure in the 1970s led to a gradual decline in these deaths, although the role of cricoid pressure remains controversial. The 1980s and beyond saw further reductions due to the implementation of rigorous preoperative fasting protocols and antacid prophylaxis. Reports from the Confidential Enquiries into Maternal Deaths during these decades reflect a marked improvement in maternal safety, with pulmonary aspiration becoming a less common cause of anaesthesia-related mortality ^27, 28^. There have been only two maternal deaths from anaesthesia related aspiration in the UK since 2000 ^29, 30^. This low incidence has been attributed to factors including improvements in training, the success of strategies such as the use of antacids, fasting protocols and finally, the increased use of neuraxial techniques ^31^. While the use of neuraxial anaesthesia reduces the risk of pulmonary aspiration compared with general anaesthesia, its precise role in the reduction in obstetric airway deaths in the United Kingdom is unclear, although it would seem self-evident that an increase in the use of neuraxial anaesthesia will lead to a reduction in deaths relating almost entirely to a complication linked to loss of consciousness. A survey of UK obstetric anaesthetic practice in the 1980s and 1990s demonstrated that although the use of neuraxial anaesthesia increased, so too did the number of emergency caesarean sections ^32^. Thus, although the proportion of emergency caesarean sections performed under general anaesthesia – the group identified as most at risk from pulmonary aspiration – decreased, the absolute number remained constant. However, fewer of these women died as a result of aspiration. Therefore, although the increased use of neuraxial anaesthesia may have prevented an increase in the number of maternal deaths from aspiration, it does not fully explain the dramatic decline of aspiration deaths in the UK. However, it is clear that avoiding general anaesthesia almost eliminates the risk of aspiration.

All the patients in our study who aspirated in association with anaesthesia had some oral intake in the 6 hours before aspirating. The practice of restricting oral intake during labour was initially introduced in the 1940s in the USA and UK in case of general anaesthesia being required for caesarean section. This was intended to prevent gastric aspiration pneumonitis, as described by Mendelson ^5^. Despite the widespread practice of restricting oral intake, debate about this topic has continued, particularly since the publication of the NICE Intrapartum Care guideline in 2007 ^11^, which recommended a less restrictive attitude to oral intake in labour in women who had not received opioids. The inherent problem is that epidural analgesia in labour predominantly employs the use of low dose local anaesthetic solutions combined with fentanyl. Opioids administered via any route can delay gastric emptying ^33, 34^. However, an effective epidural in situ also reduces the chance of general anaesthesia being needed should a caesarean section be required ^7^.

Alongside concerns about the risks of oral intake are concomitant concerns about potential detrimental effects of limiting calorific intake in labour. A randomised trial examining the effect of a light, low-fat diet versus water alone on labouring women’s metabolic profile, labour outcomes, and aspiration risk found that women who received the light diet were less likely to become ketotic compared with women who received water only. There were no differences in the duration or outcome of labour. However, mothers in the eating group had significantly larger residual gastric volumes at the time of delivery suggesting a potential higher risk of aspiration if general anaesthesia was required ^35^. Conversely, a meta-analysis that compared a strategy of less-restrictive food intake in labour with a more restrictive food intake strategy on maternal and neonatal outcomes ^36^ found that across ten studies including almost 4000 low-risk labouring women, a less-restrictive food intake policy was associated with a significantly shorter labour duration compared with a more restrictive policy and identified no cases of aspiration in either group.

Aspiration prophylaxis was not consistently used in the women who aspirated in this study. A UK survey of obstetric anaesthesia lead consultants between 1994 and 2004 found a decrease in the proportion of units using routine acid aspiration prophylaxis in all patients and an increase in the proportion using acid aspiration prophylaxis in at-risk groups only ^37^. Only 7% of respondents never used antacid prophylaxis. A large Australian study found that only 64% of women who underwent emergency caesarean section had received antacid medication ^12^. A Cochrane review examined the effectiveness of interventions at caesarean section to reduce the risk of aspiration pneumonitis found that antacids in combination with H2 antagonists were more effective than either no intervention or antacids alone in preventing low gastric pH ^38^. However, none of the studies included in that review evaluated potential harms or substantive clinical outcomes, and the quality of studies was poor. The continued use of these agents in women identified as being at higher risk of operative intervention would seem prudent and is recommended by the 2017 UK Confidential Maternal Death Enquiries Report ^30^.

A large proportion of cases had relevant medical problems including epilepsy, tracheoesophageal surgery causing swallowing problems and gastric bypass. It is unclear whether these conditions could be predisposing, or due to chance, but conditions such as these should be considered when identifying high-risk women to ensure the presence of a senior anaesthetist, giving birth in a consultant-led facility, fasting during labour and antacid prophylaxis.

Of the nine women who aspirated in association with general anaesthesia, seven received cricoid pressure. Cricoid pressure is still used routinely during obstetric general anaesthesia in the UK ^39^ and recommended for obstetric general anaesthesia ^40^. However, there is no high-quality evidence demonstrating its effect in reducing aspiration and concerns exist about its effect on visualisation of the glottis during intubation ^40^.

The women who aspirated had significantly worse outcomes, showing that pulmonary aspiration has serious sequelae. It is more difficult to draw conclusions about the fetal outcomes. Aspiration of gastric contents can increase the difficulties associated with airway management and potentially lead to hypoxia, which can affect fetal outcomes. However, major confounding factors exist for all studies involving general anaesthesia for caesarean section and neonatal outcomes and it is therefore impossible to imply causation.

This study was prospective and population-based, and did not rely on retrospective hospital records, which often do not contain the necessary information. This approach also standardised and simplified data collection. Inclusion of all consultant-led maternity units in the UK meant that no extrapolation was required to gain national-level data to estimate incidence, eliminating selection bias. However, despite this study being national and conducted over three years, we still had very limited power to identify statistically significant risk factors and were not able to investigate their relationship to the severity of the outcome.

This study has shown that the incidence of pulmonary aspiration in obstetrics is very low, despite probable increases in food and fluid intake during labour following NICE policy changes ^11^. This suggests that current anaesthetic techniques are effective in preventing aspiration events, although the high morbidity among cases emphasises the importance of prevention. The recognised risk factors of caesarean section and general anaesthesia were statistically significantly associated with aspiration, but the low power of the study meant no definite conclusions could be drawn about other probable risk factors including BMI, and lack of data for controls meant that effects of antacid prophylaxis, receipt of opioids, and anaesthetic technique could not be evaluated. Ideally, further studies would look to combine national data from several countries to gain sufficient power to draw statistically significant conclusions about risk factors and ideal management.

The results of this study suggest a reassuringly low incidence of pulmonary aspiration in obstetric anaesthetic practice; however, the complication remains a risk of general anaesthesia. Future research should focus on developing methods to accurately identify women at risk of aspiration. Gastric ultrasound has emerged as a valuable tool in obstetric anaesthetic practice, offering real-time visualisation of gastric contents and volume ^41^. This non-invasive technique allows anaesthetists to assess the risk of pulmonary aspiration by identifying patients with full stomachs, which is particularly crucial in emergency situations or when adherence to fasting guidelines is uncertain. By providing immediate and accurate information, gastric ultrasound may help stratify patients' risk and tailor anaesthetic management to enhance safety. Its use can lead to more informed decisions regarding the need for rapid sequence induction, the application of cricoid pressure, or alternative airway management strategies, ultimately reducing the incidence of aspiration-related complications in obstetric patients.

## Ethics and consent

The existing UKOSS programme has been approved by the North London REC1 (ref. 10/HO717/20) and a substantive amendment was approved on 30/04/2012 to include pulmonary aspiration in pregnancy. Consent was not required for the collection of anonymous routine data.

## Data Availability

Data cannot be shared because of confidentiality issues and potential identifiability of sensitive data as identified in the Research Ethics Committee approval. Requests to access the data can be made by contacting the National Perinatal Epidemiology Unit data access committee via general@npeu.ox.ac.uk. The Research Ethics Committee approved the study on the basis that access will only be allowed after review of the request by the UK Obstetric Surveillance System Steering Committee. The estimated response time for requests is 4 weeks. Data sharing outside the UK or the European Union may require consultation with the UK Health Research Authority. For more information, please refer to the National Perinatal Epidemiology Unit Data Sharing Policy available at: https://www.npeu.ox.ac.uk/assets/downloads/npeu/policies/Data_Sharing_Policy.pdf Preliminary incidence data from the first two years of surveillance were presented at the annual meeting of the Obstetric Anaesthetists' Association in May 2016 in Manchester, UK, and published in abstract form as: Knight M, Bogod D, Lucas DN, Quinn A and K. JJ. (2016). Pulmonary aspiration during pregnancy or immediately postpartum in the UK: a two-year national descriptive study. International Journal of Obstetric Anesthesia 26(Supplement 1): S6-S54 (Available from: https://www.obstetanesthesia.com/article/S0959-289X(16)00034-0/pdf).

## Software availability statement

All statistical analyses were performed using STATA v13.1, a commercial software. All analyses in this study can be replicated using freely available statistical software such as R.
