# Supplementary material for: Pulmonary aspiration during pregnancy or immediately postpartum in the UK: A population-based case-control study
Source: NIHR Open Res. 2025 Jan 8;5:2. [Version 1] doi: 10.3310/nihropenres.13797.1 (PMC11795022; doi:10.3310/nihropenres.13797.1)
Supplement: Peer review history

# Reviewer report: Reviewer response for version 1

**DOI:** 10.3310/nihropenres.14988.r34474

**Published:** 2025-02-08

**Authors:** Leiv Arne Rosseland, Grethe Heitmann

**Affiliations:** 1 University of Oslo, Oslo, Norway; 2 Research & Development, Division of Emergencies and Critical Care, Oslo University Hospital, Oslo, Oslo, Norway; 3 Department of Anaethesia, Østfold Hospital Trust, Fredrikstad, Norway; 4 Institute of Clinical Medicine, University of Oslo, Oslo, Oslo, Norway

Thank you for the opportunity to assess this manuscript which presents an important presentation of the risks for pulmonary aspiration in obstetric patients and how this serious anaesthetic complication has been managed in UK.

 The high-quality of the UK surveillance system for reporting and evaluating mishaps in obstetrics is a source of important knowledge relevant to the international community of obstetric anaesthetists. The objectives of this study are well presented and the methods used are appropriate and easy to understand. The main finding of this study was that the risk of pulmonary aspiration was low in pregnant women. However, as stated by the authors, this was very different a few decades ago. Owing to modern anaesthesia practices, we have succeeded in making obstetric anaesthesia safer. As the data was collected a decade ago it may limit generalisability as this development has continued. Reflexions on if, and how the practice has changed since the data was collected would help the reader evaluate the representativeness of the findings a decade after the data was collected.

 In the introduction, the authors describe the risk factors and present an overview of the topic. However, some statements and conclusions remain debatable. For example, pain may be a risk factor for delayed gastric emptying. Even if this is not evidenced by high-quality studies, it is regarded as common-sense knowledge based on the experience of generations of anaesthesiologists.

 However, a statement for which the evidence is stronger is regarding gastric emptying during pregnancy. Delayed gastric emptying during the first trimester is well documented. However, gastric emptying is not delayed in the second and third trimesters. The authors referred to Ref. #2 to support the statement that progesterone causes delayed gastric emptying. However, this is not in line with ref. #2. We question this statement and urge the authors to modify it accordingly, as updated information on the topic questions this, e.g., the recent review by Lawson et al (2024[Ref-1]) ( https://doi.org/10.1016/j.bja.2024.09.005).

 In the plain language summary, they conclude that “during the three years, 12 pregnant women in the UK experienced pulmonary aspiration when giving birth.”  When reading the entire manuscript, we were unsure if all cases occurred when giving birth. In Table 1, there was one case of missing information regarding the mode of delivery and anaesthesia, and 9 of 10 women had general anaesthesia. Please clarify if all cases occurred during delivery.

 It might seem like there is one contradiction between the results and statement in the discussion and that there is a need for clarification. It is stated: “Seven of the 12 cases (58%) were known to have had oral intake during the 6 hours preceding the aspiration event” it is also stated that 9 patients received GA. However, in the discussion part it is stated: “All the patients in our study who aspirated in association with anesthesia had some oral intake in the 6 hours before aspirating.”  It is therefore unclear if all the 9 patients who received GA, and by this aspirated in association with anesthesia, had some oral intake.

 Another statement that might be debatable is: “Opioids administered via any route can delay gastric emptying. However, an effective epidural in situ also reduces the chance of general anaesthesia being needed should a caesarean section be required”. One of the references is from 1975, and there might be more recent publications on this topic.

 A publication from 2022 found that EDA (with opioids) increased gastric emptying during labour compared to the control group. Bouvet L et al (2022 [Ref-2]) (DOI: 10.1097/ALN.0000000000004133)

 The results indicate a low risk of pulmonary aspiration when general anaesthesia is given during delivery, whereas the risk during pregnancy, in general, is zero. From a practical clinical perspective, general anaesthesia during pregnancy is safe, but general anaesthesia during delivery carries a small but significant risk of pulmonary aspiration. This also leads us to the first question: delayed gastric emptying is not a significant problem in the second and third trimesters. Women in labour experience pain and most often do not fast, both of which are reasons to expect a high risk of aspiration. As shown in Table 1, only four cases were pre-labour caesarean deliveries. As most planned C-sections are performed under regional anaesthesia, the population of planned C-sections performed under general anaesthesia is highly selected and should be interpreted with caution.  If available, more detailed information on these four cases would be interesting, such as individual risk factors, amount and type of food intake during the previous hours, indication for caesarean section, and general anaesthesia. Presumably, these four cases were emergency deliveries, but since the cases are summarised in different tables, it is difficult to get an understanding of the individual clinical situation. Presenting the data as a case series might have made this easier.

 The information that all patients who aspirated in association with anaesthesia had some food intake during the last 6 hours may be one of the most important messages: anaesthesiologists should follow general rules for fasting for all planned C-sections, even when regional anaesthesia is the chosen anaesthesia method as we never know who will need general anaesthesia due to incomplete regional anaesthesia, post-partum haemorrhage, or other unforeseen complications.

 As these cases can be interpreted as pulmonary aspiration in non-fasting patients undergoing urgent surgery, it would be interesting to compare the risk with data from a non-pregnant population. Tetsuro et al (2006 [Ref-3]) reported an incidence of 1 in 7103 GAs ( *DOI: *10.1213/01.ane.0000237296.57941.e7).

Is the work clearly and accurately presented and does it cite the current literature?

Partly

If applicable, is the statistical analysis and its interpretation appropriate?

Yes

Are all the source data underlying the results available to ensure full reproducibility?

Partly

Is the study design appropriate and is the work technically sound?

Yes

Are the conclusions drawn adequately supported by the results?

Partly

Are sufficient details of methods and analysis provided to allow replication by others?

Yes

Reviewer Expertise:

Obstetric anaesthesia

We confirm that we have read this submission and believe that we have an appropriate level of expertise to confirm that it is of an acceptable scientific standard, however we have significant reservations, as outlined above.

## References

[1] : Gastric emptying in pregnancy and its clinical implications: a narrative review. *Br J Anaesth* .2025;134(1) : 10.1016/j.bja.2024.09.005 124-167 10.1016/j.bja.2024.09.005 39443186 PMC11718367

[2] : Pregnancy and Labor Epidural Effects on Gastric Emptying: A Prospective Comparative Study. *Anesthesiology* .2022;136(4) : 10.1097/ALN.0000000000004133 542-550 10.1097/ALN.0000000000004133 35103759

[3] : The incidence and outcome of perioperative pulmonary aspiration in a university hospital: a 4-year retrospective analysis. *Anesth Analg* .2006;103(4) : 10.1213/01.ane.0000237296.57941.e7 941-7 10.1213/01.ane.0000237296.57941.e7 17000809

# Reviewer report: Reviewer response for version 1

**DOI:** 10.3310/nihropenres.14988.r34471

**Published:** 2025-02-05

**Authors:** Lisa R Leffert

**Affiliations:** 1 Yale Medicine/Yale New Haven Health System, Yale, USA

The authors present a population-based surveillance and case-controlled study utilizing the UKOSS dataset (2013-2016) to ascertain the incidence of clinically significant pulmonary aspiration during pregnancy or immediately postpartum. This is a highly worthwhile endeavor, as concern for aspiration continues to be a major consideration driving obstetric anesthesia practice. Commenced in 2005, UKOSS has been extraordinarily well positioned to study rare and severe complications of pregnancy such as this, due to its high degree of data completion and ability to be modified to address specific research questions.

 Unlike in many others, in this study aspiration is clearly defined. Patients of interest are those that have clinically significant aspiration defined as: “women at 20 weeks’ gestation or greater who had an unprotected airway while unconscious, semiconscious or paralyzed as well as a clinical history consistent with the regurgitation of stomach contents and pulmonary aspiration, such as vomiting or gastric contents seen in the oropharynx that is accompanied by signs of respiratory compromise requiring supplementary oxygen and antibiotics or levels 2/3 respiratory support without any other apparent cause. “ These patients have been compared to UKOSS controls. The results indicate that aspiration is 1) a rare event (1: 192,309 maternities) with no more than five cases in each of the three consecutive years, and 2) not fatal in this sample population. Not surprisingly, 9/12 cases were associated with general anesthesia. All the non-general anesthesia patients had comorbidities that likely put them at risk for aspiration, such as recurrent seizures or depressed consciousness. Detailed clinical data are provided as to the affected patients’ po status, premedication, and airway management. Whereas these data are interesting to consider considering our current, highly variable, premedication and liberalizing po practices, they are inconclusive due to the very small sample size. What is not provided, but would be of interest, is the incidence of aspiration in the pregnant women who received general anesthesia for cesarean delivery.

 In addition, the readers would benefit greatly from a more detailed discussion of the rationale for choosing unmatched controls from UKOSS datasets from two decades ago, rather than from the same dataset as the cases of aspiration. In the decade since this aspiration data was collected, intubation practices, including the more widespread use of videolaryngoscopy, have evolved, raising the possibility that the contemporary aspiration incidence has changed. Perhaps it is even lower.

 Finally, univariate analysis of these data showed that duration of gestation, parity, cesarean delivery, and general anesthesia for cesarean delivery were all statistically significantly associated with the occurrence of pulmonary aspiration. As noted by the authors, the total number of cases was insufficient to permit the use of a multivariable model and establishment of independent associations. Notably, both earlier gestation and nulliparity are known to be associated with cesarean delivery, which was associated with aspiration in this study. 

 In summary, these data support a very low risk of clinically significant aspiration in pregnant women, especially in those who have not received general anesthesia nor have relevant comorbidities. As the authors state, what remains is to identify those minority of pregnant patients at higher risk, so that more stringent harm reduction strategies can be focused on them.

 Respectfully Submitted,

 Lisa Leffert, MD

Is the work clearly and accurately presented and does it cite the current literature?

Yes

If applicable, is the statistical analysis and its interpretation appropriate?

Yes

Are all the source data underlying the results available to ensure full reproducibility?

Yes

Is the study design appropriate and is the work technically sound?

Partly

Are the conclusions drawn adequately supported by the results?

Partly

Are sufficient details of methods and analysis provided to allow replication by others?

Partly

Reviewer Expertise:

NA

I confirm that I have read this submission and believe that I have an appropriate level of expertise to confirm that it is of an acceptable scientific standard.

# Reviewer report: Reviewer response for version 1

**DOI:** 10.3310/nihropenres.14988.r34404

**Published:** 2025-02-04

**Authors:** Associate Professor Dominique van Dyk

**Affiliations:** 1 University of Cape Town, Cape Town, Western Cape, South Africa

Congratulations to the authors on attempting to answer lingering questions concerning the recent prevalence of, and risk factors for, an obstetric complication which has become rare, but which remains potentially very serious when it occurs.

 The introduction contextualises the historical background of aspiration in obstetric anaesthesia practice and outlines the traditional thinking surrounding pregnancy physiology and risk factors for aspiration. However, newer physiological work suggesting that stomach emptying is chiefly slowed once the pregnant person goes into labour was not mentioned. 

 The authors evidently put much thought and consultation into the study design, and I agree with the prospective case-control approach.

 There are generally some trade-offs when developing a pragmatic case reference form (CRF) - some individual case detail is invariably lost. The CRF employed sought to overcome this by asking for many data points (the relevance of some not being obvious) and was quite lengthy, but given the low incidence of aspiration, it seems to have been completed adequately when aspiration was identified, as per case definition. One addition I might have suggested to section 5b (Aspiration associated with general anaesthesia) would be laryngoscopic technique in cases where general anaesthesia was utilised- direct vs video laryngoscopy, as inadvertent oesophageal intubation may be reduced by video laryngoscopy. There was no record of difficulties associated with airway management and the need to release cricoid pressure or institute facemask ventilation. Anaesthesia technique for caesarean was not recorded by intended method- there is a difference between a "controlled" induction of general anaesthesia and emergent intubation due to cardiac arrest, on-table convulsions, or total spinal anaesthesia.  A strength of the methodology was the comprehensive coverage of all consultant-led obstetric facilities, and the cross-check with MBRRACE-UK mortality reports over the same period.

 The statistical analysis was appropriate; it was unfortunate that the small event numbers precluded multivariable logistical regression, but the authors were cognisant of this limitation.

 The authors explained why not all data could be shared and provided a pathway for accessing data upon request to the UKOSS steering committee. This was contained in the Data availability statement.

 Ultimately, the chief finding is that obstetric aspiration has become rarer in recent years. No new but plausible risk factors could be confirmed given the small case numbers. For the same reason, the description of prophylactic measures and treatment of aspiration may not reflect standard practice in the UK currently.

 It might have been interesting to discuss the much lower incidence of obstetric aspiration in this prospective study compared with a contemporary multi-centre retrospective study conducted in Israel which investigated the incidence of aspiration during caesarean delivery (see following citation).

Is the work clearly and accurately presented and does it cite the current literature?

Partly

If applicable, is the statistical analysis and its interpretation appropriate?

Yes

Are all the source data underlying the results available to ensure full reproducibility?

Partly

Is the study design appropriate and is the work technically sound?

Yes

Are the conclusions drawn adequately supported by the results?

Yes

Are sufficient details of methods and analysis provided to allow replication by others?

Yes

Reviewer Expertise:

Obstetric anaesthesia

I confirm that I have read this submission and believe that I have an appropriate level of expertise to confirm that it is of an acceptable scientific standard.

## References

[1] : Incidence and clinical impact of aspiration during cesarean delivery: A multi-center retrospective study. *Anaesth Crit Care Pain Med* .2024;43(2) : 10.1016/j.accpm.2024.101347 101347 10.1016/j.accpm.2024.101347 38278356

# Reviewer report: Reviewer response for version 1

**DOI:** 10.3310/nihropenres.14988.r34469

**Published:** 2025-02-03

**Authors:** Richard M Smiley

**Affiliations:** 1 Columbia University Vagelos College of Physicians and Surgeons, New York, USA

The authors report on 12 cases of pulmonary aspiration in obstetric patients, drawn from the UK Obstetric Surveillance System (UKOSS) form 2005-2014.  They estimated this corresponds to approximately 5.2 cases per million deliveries (based on ~ 2.3 million deliveries).  They compared the 12 cases to a cohort of 1982 controls form other UKOSS studies in an attempt to define risk factors for and associations with aspiration.

 The results of this study demonstrate the very low incidence of aspiration in modern obstetric practice.  In addition, there was only one death among the 12 patients who aspirated, and it was not attributed to the aspiration, so the number of deaths form aspiration was zero.  As might be expected, 9 of the cases were associated with general anesthesia, while the other three were associated with a seizure, a semi-conscious patient, and an unspecified cardiac issue. The association with general anesthesia, or altered consciousness is of course expected.  There were no other significant associations found with aspiration events, as might be expected with such a small number of events.

 This report is a very interesting and valuable addition to our knowledge about the true risk of aspiration in pregnancy, which has been a major concerns since Mendelson's seminal report in 1946.(1)  This study confirms the very low incidence of the event in actual modern practice, which is consistent with the zero incidence in 250,000 obstetric anesthetics in the SOAP Severe Complication Registry (SCORE) (2), and the very low number reported in recent UK triennial reports of maternal deaths. (3)

 The low incidence and even lower mortality of aspiration in the UK, a nation where “NPO” rules in labor suites are not nearly as restrictive as in the US does suggest that our restrictions on oral intake in labor may well be too strict, with fears of aspiration being based on historical practices from decades or more than half a century ago rather than reflecting the risks in contemporary practice.  

 My comments in the checkboxes about data availability and the inability to reproduce the authors results are due to the fact that this UKOSS data is not publicly available.

Is the work clearly and accurately presented and does it cite the current literature?

Yes

If applicable, is the statistical analysis and its interpretation appropriate?

Yes

Are all the source data underlying the results available to ensure full reproducibility?

Partly

Is the study design appropriate and is the work technically sound?

Yes

Are the conclusions drawn adequately supported by the results?

Yes

Are sufficient details of methods and analysis provided to allow replication by others?

Partly

Reviewer Expertise:

Obstetric anesthesia

I confirm that I have read this submission and believe that I have an appropriate level of expertise to confirm that it is of an acceptable scientific standard.

## References

[1] : The aspiration of stomach contents into the lungs during obstetric anesthesia. *Am J Obstet Gynecol* .1946;52: 10.1016/s0002-9378(16)39829-5 191-205 10.1016/s0002-9378(16)39829-5 20993766

[2] : Serious complications related to obstetric anesthesia: the serious complication repository project of the Society for Obstetric Anesthesia and Perinatology. *Anesthesiology* .2014;120(6) : 10.1097/ALN.0000000000000253 1505-12 10.1097/ALN.0000000000000253 24845921

[3] : UK Confidential Enquiry into Maternal Deaths - still learning to save mothers' lives. *Anaesthesia* .2018;73(4) : 10.1111/anae.14246 416-420 10.1111/anae.14246 29536515
